# Preparation, Characterization and Properties of Alginate/Poly(*γ*-glutamic acid) Composite Microparticles

**DOI:** 10.3390/md15040091

**Published:** 2017-04-11

**Authors:** Zongrui Tong, Yu Chen, Yang Liu, Li Tong, Jiamian Chu, Kecen Xiao, Zhiyu Zhou, Wenbo Dong, Xingwu Chu

**Affiliations:** 1 School of Material Science and Engineering, Beijing Institute of Technology, Beijing 100081, China; zrtong@163.com (Z.T.); lyang425@yeah.net (Y.L.); xiaokecen@163.com (K.X.); zhouzhiyuandy@163.com (Z.Z.); dwb194413@126.com (W.D.); 2 Department of Biochemistry and Molecular Biology, Beijing Normal University, Beijing 100875, China; 3 Taizhou Roosin Medical Co., Ltd., Taizhou 225300, China; jameschu@roosin.com (J.C.); rongxingchu@roosin.net (X.C.)

**Keywords:** alginate, poly(*γ*-glutamic acid), emulsification/internal gelation method, microparticle, swelling behavior

## Abstract

Alginate (Alg) is a renewable polymer with excellent hemostatic properties and biocapability and is widely used for hemostatic wound dressing. However, the swelling properties of alginate-based wound dressings need to be promoted to meet the requirements of wider application. Poly(*γ*-glutamic acid) (PGA) is a natural polymer with high hydrophility. In the current study, novel Alg/PGA composite microparticles with double network structure were prepared by the emulsification/internal gelation method. It was found from the structure characterization that a double network structure was formed in the composite microparticles due to the ion chelation interaction between Ca^2+^ and the carboxylate groups of Alg and PGA and the electrostatic interaction between the secondary amine group of PGA and the carboxylate groups of Alg and PGA. The swelling behavior of the composite microparticles was significantly improved due to the high hydrophility of PGA. Influences of the preparing conditions on the swelling behavior of the composites were investigated. The porous microparticles could be formed while compositing of PGA. Thermal stability was studied by thermogravimetric analysis method. Moreover, in vitro cytocompatibility test of microparticles exhibited good biocompatibility with L929 cells. All results indicated that such Alg/PGA composite microparticles are a promising candidate in the field of wound dressing for hemostasis or rapid removal of exudates.

## 1. Introduction

Wound healing is a complex process with sequential phases and demands a proper environment. Because hydrogels keep the wound surface in a moist environment, they could promote healing, according to the early work by Winters [1]. Advanced dressings should possess ideal swelling behavior to absorb exudates quickly, keep proper moisture in the covering areas and be easily removed [2,3]. Numerous super-absorbent materials have been investigated and applied for wound healing [4,5,6]. Furthermore, natural polysaccharides were studied to produce super-absorbent composites as substitutes of non-biodegradable synthetic polymers [7,8]. Among them, alginate has been widely investigated for wound dressings [9,10].

Alginate is a renewable resource and widely derived from brown algae [10,11]. Due to its non-toxicity, biocompatibility and ability to promote cell proliferation [12,13,14,15], it is widely used in tissue engineering, drug delivery and wound dressing, etc. The alginate-based wound dressings could absorb water up to several times their own weight [16,17,18]. However, wound dressings with higher swelling behavior are required as reported in the literature to help hemostasis, autolytic debridement, increased collagenase production and the moisture content of necrotic wounds. For example, Burn shield hydrogel dressing (Levtrade International) is polyurethane foam that can absorb more than 30 times its own weight in water [19,20]. A hemostatic wound dressing for severe artery hemorrhage could absorb massive water [21].

In previous studies, a natural hydrophilic polymer like poly(*γ*-glutamic acid) (PGA) was chosen for a composite with alginate instead of synthetic super-absorbent polymers. Alg/PGA composite hydrogel was prepared with improved swelling property [22]. Layered hydrogel with poly(*γ*-glutamic acid), sodium alginate and chitosan was prepared and characterized [23]. Poly(*γ*-glutamic acid) is a unique natural anionic homo-polyamide which can be produced by the fermentations by common *Bacillus* microbes [24]. It has been widely used in food and cosmetics industries due to its biocompatiblity and biodegradability. In addition, its potential application as microcapsules for drug delivery has also attracted considerable interest in recent years [25,26]. PGA is highly hydrophilic and could promote the absorption behavior of composite biomaterials by mixing it with another matrix [27].

Recently, preparation of highly hydrophilic Alg/PGA microparticles has attracted considerable attention. Suzuki et al. prepared core-shell Alg/PGA microparticles by dropping alginate solution into CaCl_2_ solution and coating the alginate microparticles with PGA [28]. Wang et al. dropped Alg/PGA mixture solution directly into CaCl_2_ solution to prepare Alg/PGA microparticles [29]. All the above works used the coacervation method to prepare Alg/PGA microparticles. In a typical coacervation process, an alginate solution is dropped into a calcium chloride solution to form microparticles by a crosslinking reaction [30]. The reaction occurs rapidly and the structure of the microparticle product is uncontrollable. In contrast, the emulsification/internal gelation technique is an effective way to prepare the microparticles with the controllable structure with the aid of the moderate crosslinking reaction in the emulsion droplets [31,32,33,34,35]. However, this method was still not used in the preparation of the Alg/PGA composite microparticles.

In the present study, the Alg/PGA composite microparticles with controllable structure were prepared using the emulsification/internal gelation method, and then the conditions to control the structure and properties of the composite microparticles were studied and discussed. The emulsification/internal gelation method could promote the properties and widen the application of these microparticles.

## 2. Results and Discussion

### 2.1. Characterization of Alg/PGA Composite Microparticles

**Figure 1:**
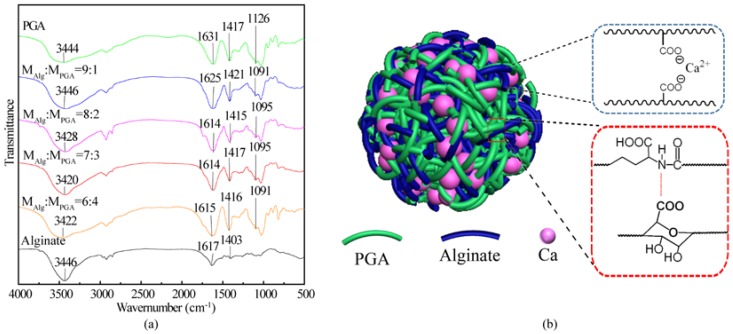
(**a**) FT-IR spectra of alginate, PGA and composite microparticles with various contents; (**b**) double-network structure scheme of the composite microparticle.

Figure 1 shows the FT-IR spectra of alginate, PGA and Alg/PGA microparticles prepared under different conditions. The stretching vibrations of the O–H in alginate and PGA led to broad absorption bands centered at 3446 cm^−1^ and 3444 cm^−1^ respectivley [36]. The corresponding absorption band of microparticles shifted to 3420 cm^−1^, indicating the presence of hydrogen bonds between alginate and PGA.

The asymmetric stretching vibration peak of the C=O in the carboxylate (COO^−^) was observed at 1631 cm^−1^ and belongs to PGA. The corresponding peak was blue-shifted in the spectrum of the composite microparticles due to the chelating action between the COO^−^ and Ca^2+^. A maximum shift to 1614 cm^−1^ was observed in the spectra of the composite microparticles prepared with m_Alg_:m_PGA_ = 7:3 and 8:2, indicating that the strongest chelation interaction occurred in these composites. Alginate is a water-soluble anionic polysaccharide consisting of repeat units of mannuronic acid and glucuronic acid. The electrostatic interaction occurs between the carboxylate groups of alginate and high valent cations [35]. The chelating interaction between Ca^2+^ and carboxylate groups of glucuronic acid and PGA is also a possible interaction in the emulsification/internal gelation method.

The absorption peak at 1126 cm^−1^ in the spectrum of PGA was assigned to the C–N stretching vibration of the secondary amine. The corresponding peak of the composite microparticles was shifted dramatically to 1095 cm^−1^ due to a strong electrostatic interaction between the amide group of PGA and the carboxylate group of alginate. In full, a double network structure (Figure 1b) was formed in the Alg/PGA microparticles via two kinds of interaction: the ion chelation between Ca^2+^ and carboxylate groups of PGA and Alginate and the electrostatic interaction between the secondary amine group of PGA and carboxylate groups of alginate and PGA.

**Figure 2:**
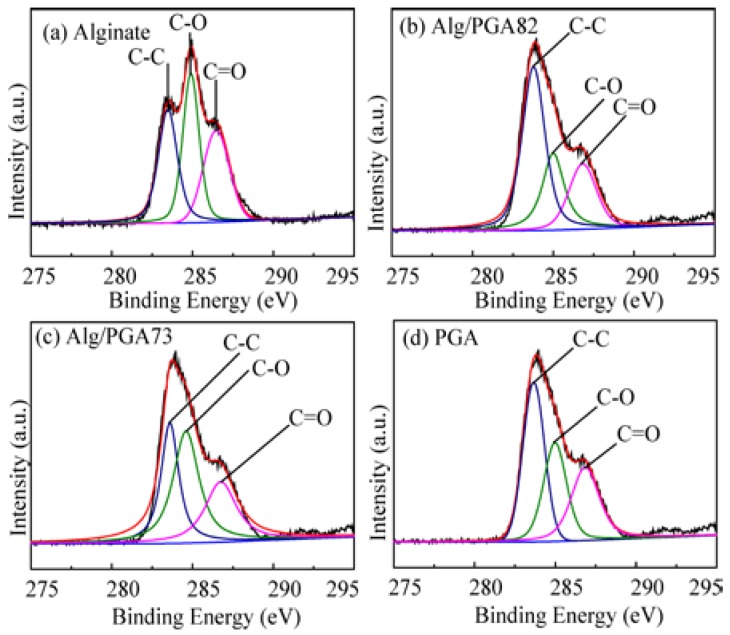
XPS C1s spectra of alginate (**a**), Alg/PGA82 (composite microparticles whose mass ratio is m_Alg_:m_PGA_ = 8:2) (**b**), Alg/PGA73 (composite microparticles whose mass ratio is m_Alg_:m_PGA_ = 7:3) (**c**) and PGA (**d**).

To further investigate the structure of the Alg/PGA microparticles, their surface chemical bonds were determined with the wide scan XPS. As shown in Figure 2, the binding energy (B.E.) peaks at 283.4, 284.9 and 286.7 eV can be assigned to C–C, C–O and C=O, respectively [37]. The relative content ratio of C to O (C/O) in the microparticles is higher than that in Alg and is increased with the increase of the PGA content in feeding materials (Table 1). In addition, the content of C=O group in the composite microparticles is significantly higher than that in alginate and is also increased with the increase of PGA content in the microparticles. These results indicate the presence of PGA and Alg in the composite microparticles.

**Figure 3:**
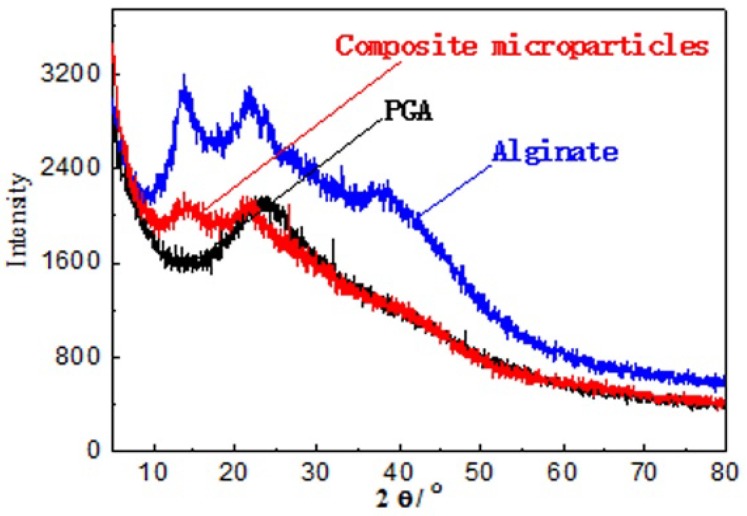
XRD patterns of Alginate, PGA and composite microparticles.

Figure 3 shows the X-ray diffraction (XRD) patterns of Alg, PGA and Alg/PGA microparticles. Alg exhibited two typical crystalline diffraction peaks at 13.73° and 21.71°. One crystalline diffraction peak was found at 21.43° in the XRD spectrum of PGA [38,39]. The Alg/PGA composite microparticles exhibited two broad diffuse diffraction peaks centered at 14.10° and 26.61°, indicating its poor crystallinity after the gelation. This can be explained by the fact that the original polymer crystal structures of Alg and PGA were destructed during the formation of the full-interpenetrating composite polymer, resulting in decreased lattice density. Meanwhile, the aggregation degree of the microparticles was increased due to the chelation between the COO^−^ and Ca^2+^ and electrostatic interaction between polyelectrolytes. Therefore, the crystalline diffraction peaks of the composite were diffused and a network structure was established in the composite microparticles.

### 2.2. Morphology of the Alg/PGA Composite Microparticles

**Figure 4:**
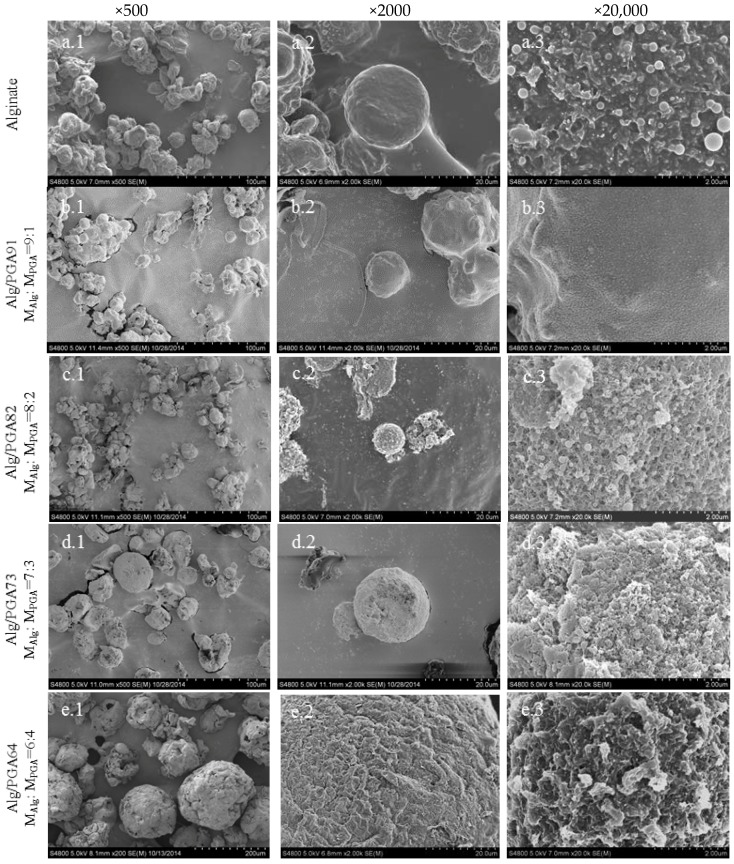
Morphology of the Alg/PGA Composite Microparticles. Note: SEM images of various microparticles at 500, 2000 and 20,000 magnification scales (**a**–**e** belongs to alginate microparticles and Alg/PGA composite microparticles with mass ratio is m_Alg_:m_PGA_ = 9:1, 8:2, 7:3, 6:4 respectively).

The surface morphology of the composite microparticles prepared with different mass ratios of Alg and PGA were observed by SEM (Figure 4) and particles size distributions were shown in Supplementary Figure S1 and Table S1. A fluctuating microparticle size (Mean size from 148.8 ± 13.0 to 9.4 ± 0.5 μm) can be observed in Figure 4a–e (×500) with an increase of PGA content. According to Supplementary Figure S1 and Table S1, the microparticle sizes decreased at first, increased later with increasing PGA content, and reach the turning point when m_Alg_:m_PGA_ = 8:2 (9.4 ± 0.5 μm). The porous structure of composite microparticles is obvious in Figure 4 (×20,000) with the involvement of PGA compared with alginate microparticles. Relatively concentrated pores with nanoscale microparticles prepared with m_Alg_:m_PGA_ = 8:2 could be found.

Higher feeding ratio of PGA leads to bigger composite microparticles and more irregular particle surface. Clusters on the surface of microparticles prepared with m_Alg_:m_PGA_ = 6:4 were also detected. When compared with other types of microparticles, the PGA microparticles contain rare and larger pores.

In all, the fluctuating changes of microparticles with PGA content increase and different porous surfaces could be concluded via SEM images. The microparticles prepared with m_Alg_:m_PGA_ = 8:2 are smallest, and the microparticles prepared with m_Alg_:m_PGA_ = 8:2 and 7:3 own concentrated pores on their surfaces.

### 2.3. Swelling Behavior of Alg/PGA Composite Microparticles

**Figure 5:**
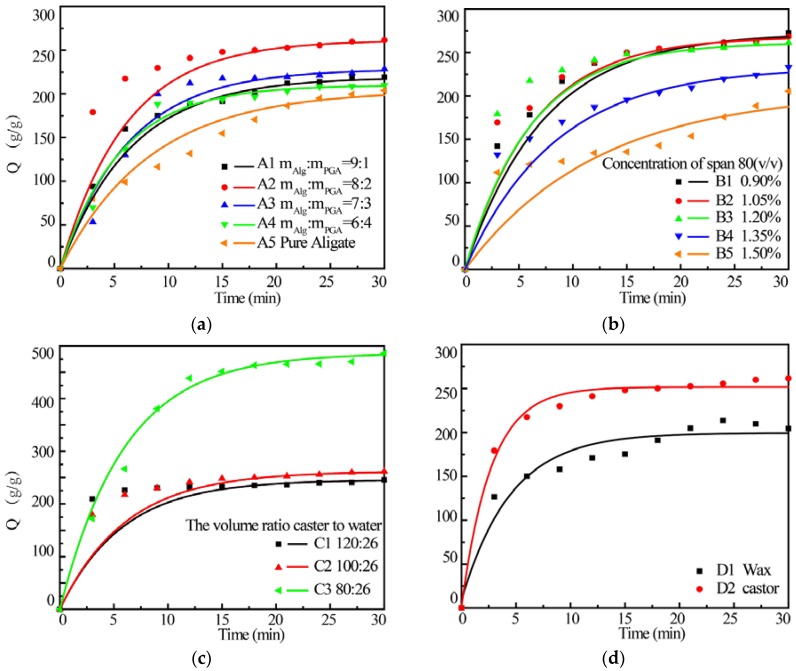
Effect of feeding ratio (**a**), concentration of span 80 (**b**), W/O ratio (**c**) and oil phase (**d**) on the swelling behavior of Alg/PGA composite microparticles.

Figure 5 shows the swelling behavior of various composite microparticles prepared under different conditions. The swelling kinetics of microparticles was fitted by Voigt model and the kinetic parameters are listed in Table 2. It can be seen that both maximum water up-take ratio (*σ*_0_/*E*) and swelling rate (*k_i_*) were increased with the increase of the PGA amount in the composite and reached the maximum in the composite A2 prepared with m_Alg_:m_PGA_ = 8:2. Further increasing PGA content leads to decreased *σ*_0_*/E* and *k_i_*. Relatively low *τ*_0_, *t_c_* of composite A2 could also represent the relatively fast swelling rate. However, the maximum water up-take ratio and swelling rate of sample A4 prepared with a m_Alg_:m_PGA_ = 6:4 are still higher than those of pure alginate microparticles.

As discussed above, PGA was incorporated into the composite microparticles and the interaction between PGA and alginate is very strong. The swelling capacity and rate of the composite were increased with the increasing of PGA content in the microparticles. Therefore, the better swelling behavior of the composite microparticles is largely attributed to a good hydrophility of PGA. In addition, the porous surface of the microparticles (Figure 4) can increase the contact area between the microparticles and water, which promotes the water absorption rate due to the capillary water absorption effect. However, the microparticles with extreme high PGA content become larger and thus the specific area of the microparticles decreased sharply, leading to low swelling capacity and rate. The maximum swelling capacity and rate were obtained in the Alg/PGA composite prepared with m_Alg_:m_PGA_ = 8:2.

The emulsifier can influence the formation of the composite microparticles by reducing the surface tension of the system and promoting the stability of water/oil (W/O) emulsion [31]. As shown in Figure 5b, both water absorption ratio and swelling rate were slightly decreased as the concentration of non-ionic surfactant span 80 was increased to 1.2%. Theoretically, the size of composite microparticle is decreased with the increase of the emulsifier concentration due to the improved dispersion. However, the extremely high concentration of span 80 may cause decreased emulsions and increased composite drop size, leading to decreased swelling capacity and rate [32,40]. Therefore, concentration of span 80 was optimized as 1.2% (*v*/*v*) for the preparation of composite microparticles with stable structure and proper swelling behavior.

The effects of the oil to water ratio in the reaction medium on the swelling behavior were further studied. The results indicate sample C5 prepared in a medium with V_castor_:V_water_ = 80:26 showed the fastest absorption rate and highest water absorption ratio at the same time (Figure 5c and Table 2). The oil content can also significantly affect the droplet size of the composite. Higher oil phase content leads to a reduction in specific surface area and an increased droplet size, resulting in undesired swelling behavior [41].

The swelling behaviors of the microparticles prepared with different dispersing phases were also investigated. The water absorption ratio and rate of the composite microparticles prepared in castor oil are higher than those of the microparticles prepared in wax. It has been reported that the emulsion size and composite particle size decreased with the increase of the reaction medium viscosity [42]. Therefore, the higher viscosity of castor oil leads to smaller emulsion, higher specific surface areas and better swelling behavior of the composite microparticles.

In all, the optimum conditions for preparation of Alg/PGA composite microparticles with excellent swelling behavior are a reaction medium with castor oil as oil phase and V_castor_:V_water_ = 80:26, 1.2% (*v*/*v*) span 80 as emulsifier and the feeding ratio of m_Alg_:m_PGA_ is 8:2.

### 2.4. Thermal Stability of Alg/PGA Composite Microparticles

The TGA and DTG curves of alginate, PGA and the composite microparticles, prepared under different conditions, are shown in Supplementary Figure S2. The thermal degradation process of alginate occures in three stages. At the first stage, the coordinated water in alginate was removed due to the dehydration and breaking of the glycosidic bonds at temperatures below 200 °C. During the second stage, in the temperature range of 200–280 °C, alginate skeleton was fractured. Further increasing the temperature causes the degradation of carboxylate group and the release of CO_2_ [43,44]. The weight loss of PGA at temperatures lower than 250 °C may be attributed to the desorption of free water and surface hydroxyl groups. The weight loss at temperatures higher than 250 °C is caused by decomposition [45]. Similarly, the thermal degradation process of Alg/PGA composite microparticles occurs in three stages in the temperature ranges of 50–200 °C, 200–280 °C and 280–450 °C respectively. The parameters at each thermal degradation stage are listed in Table 3. The weight loss at the first stage is attributed to the water loss. The primary decomposition of alginate skeleton and degradation of PGA occurred during the second stage. The third degradation stage can be assigned to the secondary decomposition of alginate skeleton and degradation of PGA. The weight loss of the composite microparticles in the temperature range of 200–280 °C was lower than that of Alg at the corresponding temperature. The temperatures required for 5% and 50% weight loss in the composite microparticles are higher than those required for the corresponding weight loss in Alg and lower than those required for the corresponding weight loss in PGA. Among all the composites, the microparticles prepared with m_Alg_:m_PGA_ = 8:2 required the highest temperatures for 5% and 50% weight loss.

Compared with alginate, the thermal stablity of composite microparticles was promoted by the ionic crosslinking, hydrogen bonding and the electrostatic binding between the polyelectrolytes. It can be concluded that the introduction of PGA promotes the thermal stablity of the composite microparticles and the composite with strongest intermolecular interactions shows the highest thermal stablity.

### 2.5. In Vitro Cytotoxicity and Compatibility

**Figure 6:**
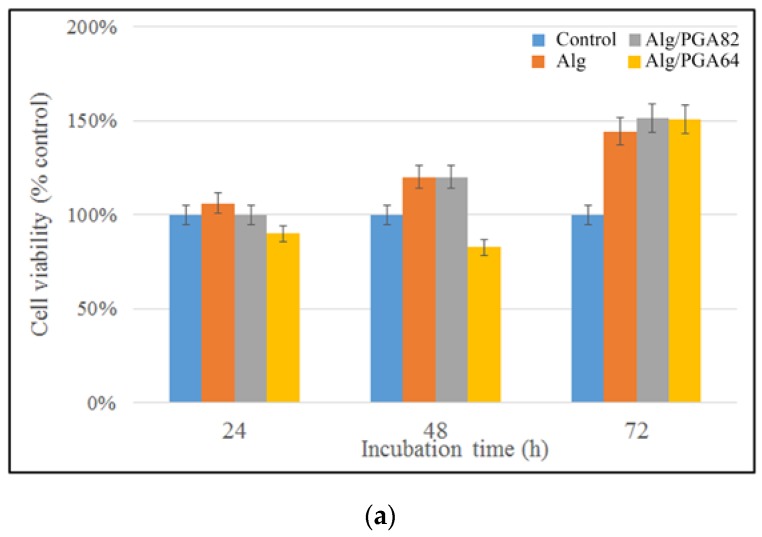

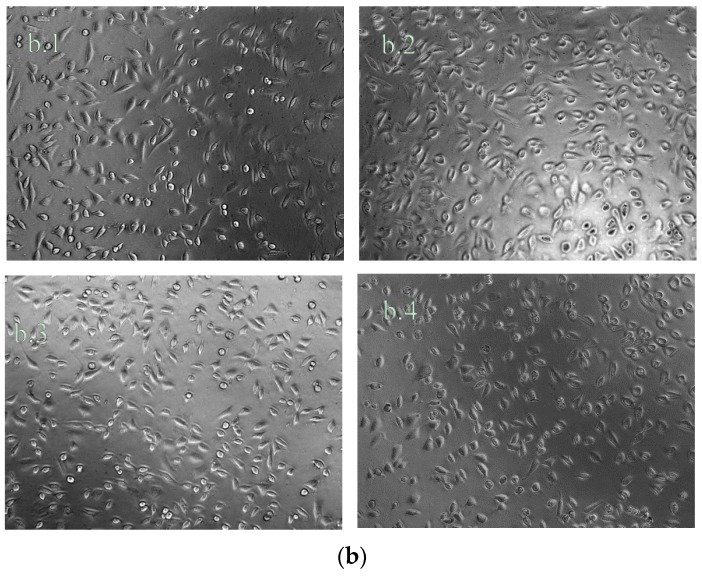
(**a**) Cytotoxicity assay of leach liquors. (**b**) Micrographs of cells cultured in leach liquors of samples Alg (**b.1**), Alg/PGA82 (**b.2**), Alg/PGA64 (**b.3**) and control group (**b.4**). Note: Samples Alg, Alg/PGA82 and Alg/PGA64 represent alginate microparticles, composite microparticles prepared with m_Alg_:m_PGA_ = 8:2 and composite microparticles prepared with m_Alg_:m_PGA_ = 6:4 respectively.

Cytotoxicity is one of the most important methods for biological safety evaluation [45]. The cytotoxicity of Alg/PGA composite microparticles was evaluated with cellular morphology and relative cell viability (RCV). As shown in Figure 6, the cytotoxicity of the leach liquors of the composite microparticles are 0 grade or 1 grade according to the RCV after 24, 48 and 72 h cultures, which means that the cytotoxicity evaluation is valid. There was no significant difference between experimental groups and control group after 24 and 48 h cultures (*p* > 0.05). After the 72 h culture, obvious enhanced cell viability of all three experimental groups was observed compared with control group according to the CCK-8 assay. As shown in Figure 6b, no pronounced cell debris or changes in morphology, such as cell lysis, loss of spindle shape or detachment from the bottom, were observed in the samples. The above results confirm good cytocompatibility of the leach liquors of the composite microparticles.

## 3. Materials and Methods

### 3.1. Materials

Sodium Alginate (NaAlg), with a viscosity of 200 ± 20 Mpa·S, was purchased from Aladdin Industrial Corporation (Los Angeles, CA, USA). Span 80 was provided by Tianjin Guangfu Fine Chemical Research Institute (Tianjin, China). Castor oil was purchased from Tianjin Chemical Reagent Factory (Tianjin, China). Liquid paraffin was acquired from Beijing Chemical Factory (Beijing, China). All of these materials were analytical grade. PGA (>92.0%) with a molecular weight of 1000 kDa was purchased from Nanjing Saitaisi Biotechnology Co., Ltd. (Nanjing, China), MEM-EBSS (Minimum Essential Medium; Sigma–Aldrich, St. Louis, MO, USA).

### 3.2. Preparation of Alg/PGA Composite Microparticles

A 6 mL suspension of 0.5% (*w*/*v*) CaCO_3_ and 1.0% (*w*/*v*) polyvinylpyrrolidone (PVP) was dispersed uniformly into 20 mL 2% (*w*/*v*) Alg/PGA aqueous solution with specific weight ratio between Alg and PGA. The mixture was added into 50 mL castor oil containing 1.2% (*v/v*) Span 80 under stirring and was mechanically stirred for 15 min. Another 50 mL castor oil containing 2% (*v/v*) acetic acid was added slowly into the mixture prepared above. The reaction mixture was stirred at 40 °C for 1 h, kept stand for 24 h and vacuum filtered. The residue was washed with 200 mL ethanol at 60 °C for 2 h and dried at 60 °C for 24 h.

### 3.3. Characterization of Alg/PGA Composite Microparticles

FTIR spectra were obtained by a Thermo-Nicolet NEXUS 470 Spectroscopy (ThermoFish, Waltham, MA, USA) equipped with a KBr beam splitter. The test samples were prepared as KBr pellets.

X-ray Photoelectron Spectroscopy (XPS) spectra were obtained by a PHI QUANTERA-II instrument (Ulvac-PHI Inc. Chigasaki, Kanagawa, Japan) equipped with a monochromatized Al KRX-ray source operated at 25 W and 15 kV. For wide-scan spectra, an energy range of 0–1100 eV was used with pass energy of 280.00 eV and a step size of 1.00 eV. High-resolution spectra were collected at 26.00 eV pass energy using a step size of 0.025 eV. The XPS results were interpreted as binding energies that were further fitted in a nonlinear least squares curve fitting program (XPS-peak-41 software).

XRD diffractograms were recorded in the 2θ range of 5.0°–80.0° on a Rigaku D/Max-1200 instrument (Rigaku, Tokyo, Japan) equipped with Ni-filtered Cu Kα radiation (40 kV, 40 mA) to determine the crystallinity of Alg/PGA composite microparticles.

SEM was used to examine the structure and surface morphology of the produced microparticles. Microparticles were dusted onto a double-sided tape on an aluminum stub, coated with a gold layer using a gold sputter coater and imaged on an S-4800 SEM instrument (Hitachi, Tokyo, Japan) with a 5 kV electron beams.

### 3.4. Determination of the Swelling Behavior of Composite Microparticles

The swelling behavior of the composite microparticles was investigated by the filtering bag test method. A certain amount of sample was put in a nylon bag and immersed into the liquid to be absorbed at room temperature. The mass of the swollen sample was weighed every 3 min after the excess water removed. The water uptake ratio (*Q*) of the microparticle was calculated as the following:

$$Q=((W_{1}-W_{0}-W_{2})/W_{0})\times 100\%$$

where, *W*_1_ is the weight of the test sample and bag at a given swelling time, *W*_2_ is the weight of nylon bag and *W*_0_ is the initial weight of the sample.

The swelling process of the composite microparticles is similar to the creeping response of polymer. It is caused by the combination of hydrophilic interactions, the repulsion force between the anions and the osmotic pressures between inside and outside of the networks. Therefore, the swelling kinetic parameters of the composite microparticles can be fitted by the Voigt model [46]. Assuming that the force *σ*_0_ is applied to the model at time *t*_0_ and the corresponding response *ε* was produced at time *t*, the model can be expressed as the following:

$$\varepsilon \;(t)=\sigma_{0}/E\{1-\exp[(t_{0}-t)/\tau_{0}]\}=\varepsilon \;(\infty )\{1-\exp[(t_{0}-t)/\tau_{0}]\}$$

where, *τ*_0_ is the relaxation time that is theoretically inversely proportional to the swelling ratio of the microparticles and *E* is Young’s modulus that represents the resistance to deformation. *σ*_0_*/E* equals to *ε_(∞)_*, representing the maximum swelling ratio. The slope of forward straight part of the curve (*k_i_*) with *Q* = 0.7*ε_(∞)_* and the time (*t_c_*) when the swelling becomes slow can be calculated accordingly. The swelling rate is directly proportional to *k_i_* and inversely proportional to *t_c_*.

### 3.5. Thermal Stability Study of Composite Microparticles

Thermogravimetric analysis (TGA) was carried out on a DTG-60 Thermogravimetry Analyzer (SHIMADZU, Kyoto, Japan). Samples with weights of 3–5 mg were heated from 50 to 450 °C with heating rate of 10 °C /min in a nitrogen atmosphere with the flow rate of 50 mL/min.

### 3.6. In Vitro Cytotoxicity and Compatibility

The preliminary investigation of indirect in vitro cytotoxicity and cytocompatibility with L929 cells (provided by Cell Bank of Chinese Academy of Sciences, Beijing, China) was performed. The microparticles were sterilized by irradiation with ^60^Co (at the room temperature, dose rate was 100 Gy/min and the irradiation dose was 15 kGy), and leached in MEM-EBSS for 24 to swell completely. The supernatant was leached at polymer concentration of 1/400 (g/mL) and collected after centrifuged at 3000 r/min for 5 min for further use. The leach liquor of the microparticles was diluted with the cell culture fluid to final concentration of 50% for CCK-8 assays.

The culture was maintained in the incubator (Zhongxing Co. Ltd., Beijing, China) at 37 °C under a wet atmosphere containing 5% CO_2_. MEM-EBSS supplemented with 10% horse serum, together with non-essential amino acid was used as cell culture fluid. The cell suspension was injected into a 96-well culture plate with 6 wells for each group and 5 × 10^4^ cells in each well and cultured in the incubator for 4 h. Then 100 μL of the leach liquors and diluted leach liquors with the concentration of 50% were respectively added into the wells of the experimental groups and 100 μL of the control solutions were respectively added to the control groups. The cells were then cultured in the incubator for 48 h. Micrographs of cell cultured in leach liquors were taken before the addition of CCK-8 with Axio Vert A1 light microscope. 20 μL CCK-8 soultion was injected into every well, then kept in incubator for 2 h. The absorbance of each well at 570 nm was determined with a POLARstar Omega analyzer. The relative cell viability (RCV) was calculated with the following Equation:

$$\mathrm{RCV}=\frac{{\left[\mathrm{Abs}\right]}_{Sample}}{{\left[\mathrm{Abs}\right]}_{Control}}\times 100\%$$

And the average value was reported.

The cytotoxicity was evaluated according to the following standard [47]: the cytotoxicity is zero grade for RCV higher than 100%, 1st grade for RCV in the range of 75%–99%, 2nd grade for RCV in the range of 50%–74%, 3rd grade for RCV in the range of 25%–49%, 4th grade for RCV in the range of 1%–24%, and 5th grade for RCV = 0%. The 1st grade or less cytotoxicity is acceptable. If the cells showed 2nd grade response to the polymer, the cell morphology should be analyzed for comprehensive evaluation. The response level equal to or higher than 3rd grade indicates that the polymer is off specification for cytotoxicity evaluation.

### 3.7. Statistical Analysis

Statistical analysis was performed using SPSS version 20 (Statistical Package). Value of *p* ≤ 0.05 was considered to be significant. All the values are expressed as means ± SD.

## 4. Conclusions

Alg/PGA composite microparticles with controllable structure were successfully prepared through the emulsification/internal gelation method and then characterized. Inside the microparticles, a double network structure was formed by ion-chelating interaction between carboxylate groups and Ca^2+^ and electrostatic interaction between carboxylate groups and secondary amine group. The swelling behavior of microparticles was promoted by the introduction of PGA. The conditions for the preparation of Alg/PGA microparticles with excellent swelling behavior were optimized. These Alg/PGA microparticles could absorb hundreds of times their weight in water. Aporous surface could be observed on composite microparticles. The morphological structure could also influence the variation of swelling behavior. The composite microparticles also showed better thermal stability compared with alginate. Alg/PGA composite microparticles also possess good biocompatibility. Alg/PGA composite microparticles are promising candidates in the field of wound dressing for hemostasis as well as for rapidly removing exudates.

## Figures and Tables

**Table 1:** Relative content of O and C in alginate, PGA and microparticles.

Samples	Relative Content Ratio of C/O	C–O		C=O	
		B.E. (eV)	Relative Content	B.E. (eV)	Relative Content
Alginate	1.6	531.37	81.1%	529.84	18.9%
Alg/PGA82 (m_Alg_:m_PGA_ = 8:2)	1.9	531.40	69.7%	530.00	30.3%
Alg/PGA73 (m_Alg_:m_PGA_ = 7:3)	2.1	531.45	51.4%	530.19	48.6%
PGA	2.2	531.50	45.1%	530.25	54.9%

**Table 2:** Swelling kinetic parameter of various microparticles.

Group	Sample	(*σ_0_*/*E*)/g·g^−1^	*τ_0_*/min	*k_i_*/g·min^−1^	*t_c_*/min
A	A1	219.3	6.5	19.5	7.9
	A2	261.6	6.1	25.0	7.3
	A3	228.6	6.3	22.8	7.0
	A4	210.0	5.5	21.6	6.8
	A5	204.1	8.3	14.2	10.0
B	B1	272.6	7.3	21.9	8.7
	B2	268.3	6.3	24.8	7.6
	B3	261.6	6.1	25.0	7.3
	B4	233.3	8.3	16.4	10.0
	B5	205.5	12.3	9.81	14.7
C	C1	236.0	1.4	25.9	6.4
	C2	261.6	6.1	25.0	7.3
	C3	482.7	6.0	47.8	7.1

**Table 3:** Thermogravimetric analysis of SA, PGA and various SA/PGA composite microparticles from TG-DTG analysis.

Sample	Stage	Temperature Range (°C)	T_max_ (°C)	Weight Loss (%)	Weight Loss 5% (°C)	Weight Loss 50% (°C)
PGA	1	50–250	55	11.84	155	Above 450
	2	250–450	345	35.74		
Alginate	1	50–200	184	12.03	77	287
	2	200–280	252	36.20		
	3	280–450	361	10.71		
m_Alg_:m_PGA_ = 9:1	1	50–200	116	12.57	80	312
	2	200–280	263	31.32		
	3	280–450	405	15.22		
m_Alg_:m_PGA_ = 8:2	1	50–200	79	9.04	104	341
	2	200–280	252	32.01		
	3	280–450	349	19.62		
m_Alg_:m_PGA_ = 7:3	1	50–200	79	10.67	88	336
	2	200–280	250	32.06		
	3	280–450	354	17.20		
m_Alg_:m_PGA_ = 6:4	1	50–200	122	11.56	75	338
	2	200–280	279	26.94		
	3	280–450	319	27.95		

## Supplementary Materials

The following are available online at www.mdpi.com/1660-3397/15/4/91/s1, Figure S1: Particles size distribution of alginate microparticles and various composite microparticles (Alg/PGA91, Alg/PGA82, Alg/PGA73, Alg/PGA64 represent composite microparticles whose mass ratio is m_Alg_:m_PGA_ = 9:1, 8:2, 7:3, 6:4 respectively), Figure S2: TG (a) and DTG (b) curves of Alginate, PGA and Alg/PGA microparticles prepared at different weight ratio, Table S1: Size distribution statistic data of various microparticles.
