# Supplementary material for: Preparation, Characterization and Properties of Alginate/Poly(γ-glutamic acid) Composite Microparticles
Source: Mar Drugs. 2017 Apr 11;15(4):91. doi: 10.3390/md15040091 (PMC5408237; doi:10.3390/md15040091)
Supplement: Supplementary file 1 [file marinedrugs-15-00091-s001.pdf]

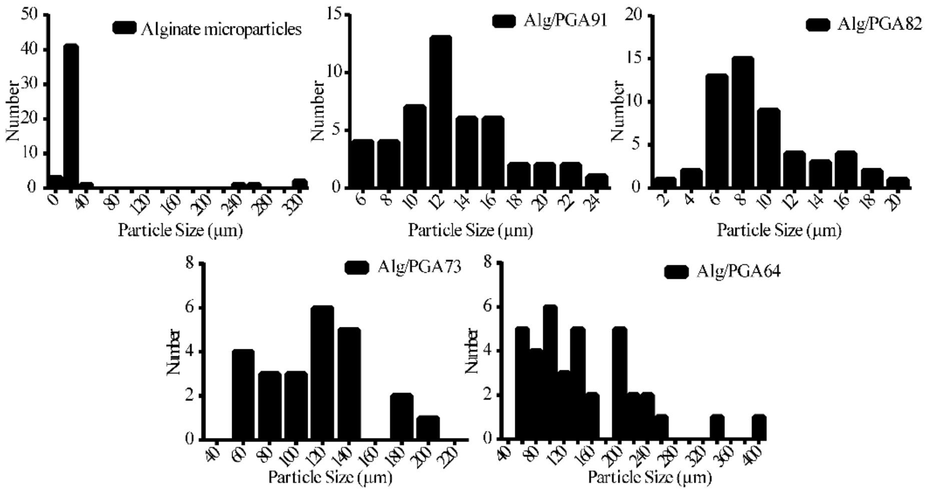

Figure S1. Particles size distribution of alginate microparticles and various composite microparticles (Alg/PGA91, Alg/PGA82, Alg/PGA73, Alg/PGA64 represent composite microparticles whose mass ratio is m_{Alg}: m_{PGA} = 9:1, 8:2, 7:3, 6:4 respectively.)

Table S1. Size distribution statistic data of various microparticles

	**Alginate**	**Alg/PGA91**	**Alg/PGA82**	**Alg/PGA73**	**Alg/PGA64**
**Mean (μm)**	39.0	13.0	9.4	117.2	148.8
**Std. Deviation (μm)**	74.0	4.2	3.8	38.0	78.9
**Std. Error of Mean (μm)**	10.6	0.6	0.5	7.8	13.0
**25% Percentile (μm)**	12.2	10.0	6.7	83.6	89.6
**Median (μm)**	17.5	12.6	8.5	120.0	132.2
**75% Percentile (μm)**	22.9	15.4	11.2	139.8	204.3
**Maximum (μm)**	311.4	23.4	19.2	209.4	390.9
**Lower 95% CI of mean (μm)**	17.8	11.7	8.4	101.1	122.5
**Upper 95% CI of mean (μm)**	60.3	14.2	10.5	133.2	175.1

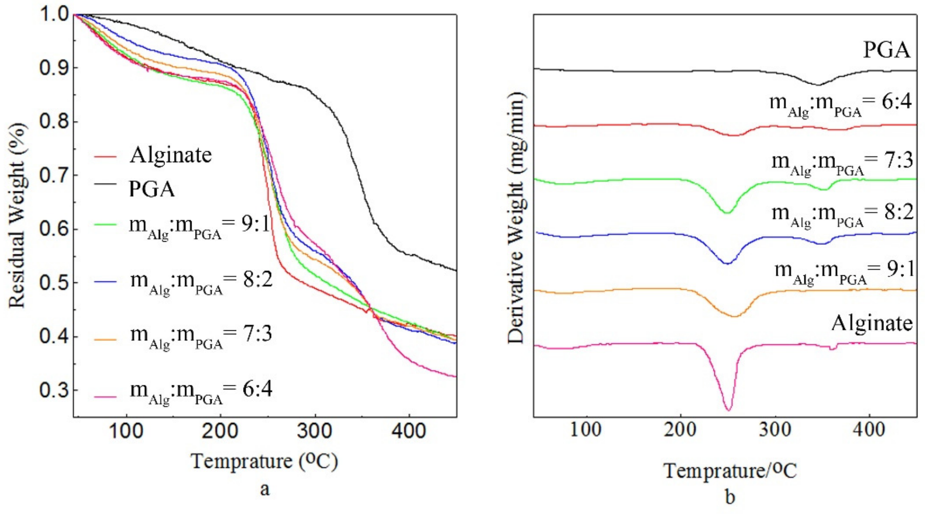

Figure S2. TG (a) and DTG (b) curves of Alginate, PGA and Alg/PGA microparticles prepared at different weight ratio.
